# Supplementary material for: In situ activation of flexible magnetoelectric membrane enhances bone defect repair
Source: Nat Commun. 2023 Jul 10;14:4091. doi: 10.1038/s41467-023-39744-3 (PMC10333320; doi:10.1038/s41467-023-39744-3)
Supplement: Supplementary file 1 — Supplementary Information [file 41467_2023_39744_MOESM1_ESM.pdf]

Supplementary Information

In situ activation of flexible magnetoelectric membrane enhances bone defect repair

Wenwen Liu[1], Han Zhao[1], Chenguang Zhang[2], Shiqi Xu[3], Fengyi Zhang[4], Ling Wei[5], Fangyu Zhu[1], Ying Chen[6], Yumin Chen[1], Ying Huang[1], Mingming Xu[1], Ying He[1], Boon Chin Heng[7], Jinxing Zhang[8], Yang Shen[9], Xuehui Zhang[10]*, Houbing Huang[2]*, Lili Chen[11]* and Xuliang Deng[1]*

These authors contributed equally: Wenwen Liu, Han Zhao, Chenguang Zhang.

Affiliations

[1] Department of Geriatric Dentistry, Peking University School and Hospital of Stomatology, Beijing, P. R. China

[2] Hospital of Stomatology, Guanghua School of Stomatology, Sun Yat-sen University, Guangzhou, P. R. China

[3] School of Materials Science and Engineering & Advanced Research, Institute of Multidisciplinary Science, Beijing Institute of Technology, Beijing, P. R. China

[4] Department of Orthopedics, The Second Xiangya Hospital, Central South University, Changsha, P. R. China

[5] Third Clinical Division, Peking University School and Hospital of Stomatology, Beijing, P. R. China

[6] Department of Prosthodontics, The First Clinical Division, Peking University School and Hospital of Stomatology, Beijing, P. R. China

[7] Central Laboratory, Peking University School and Hospital of Stomatology, Beijing, P. R. China

[8] Department of Physics, Beijing Normal University, Beijing, P. R. China

[9] State Key Laboratory of New Ceramics and Fine Processing Department of Materials Science and Engineering Tsinghua University, Beijing, P. R. China

[10] Department of Dental Materials & Dental Medical Devices Testing Center, Peking University School and Hospital of Stomatology, Beijing 100081, PR China.

[11] Department of Stomatology, Union Hospital, Tongji Medical College, Huazhong University of Science and Technology, Wuhan, P. R. China

Corresponding authors

Correspondence to Xuehui Zhang, Houbing Huang, Lili Chen, or Xuliang Deng.

*Emails: zhangxuehui@bjmu.edu.cn; hbhuang@bit.edu.cn; lily-c1030@163.com; kqdengxuliang@bjmu.edu.cn

Table of Contents

Supplementary Figure 1-19..3

Supplementary Table 1-2..22

Supplementary References 24

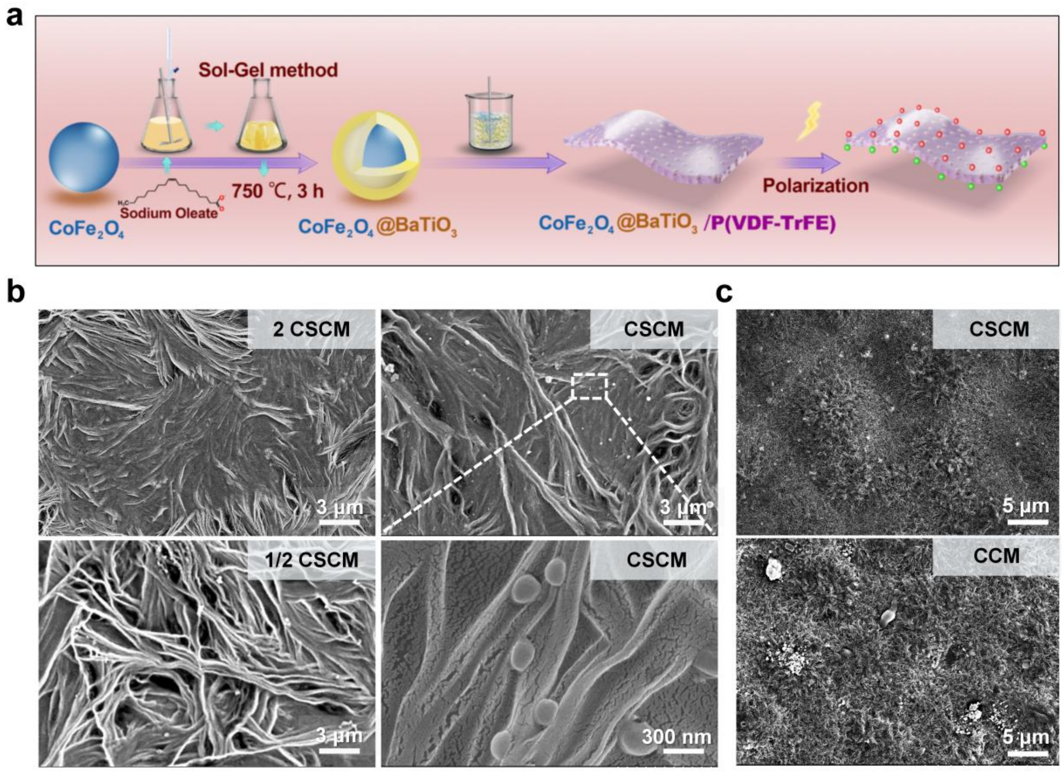

Supplementary Fig. 1 The fabrication process and characterization of the basic properties of composite membranes.

a, Fabrication process of CFO@BTO core-shell particles and CFO@BTO/P(VDF-TrFE) membrane. **b,** SEM images of 2 CSCM, CSCM and 1/2 CSCM. An enlarged view of the interior of CSCM is shown at the lower right. **c,** SEM images of the surface of CSCM and CCM. At least three times of experiments were repeated independently.

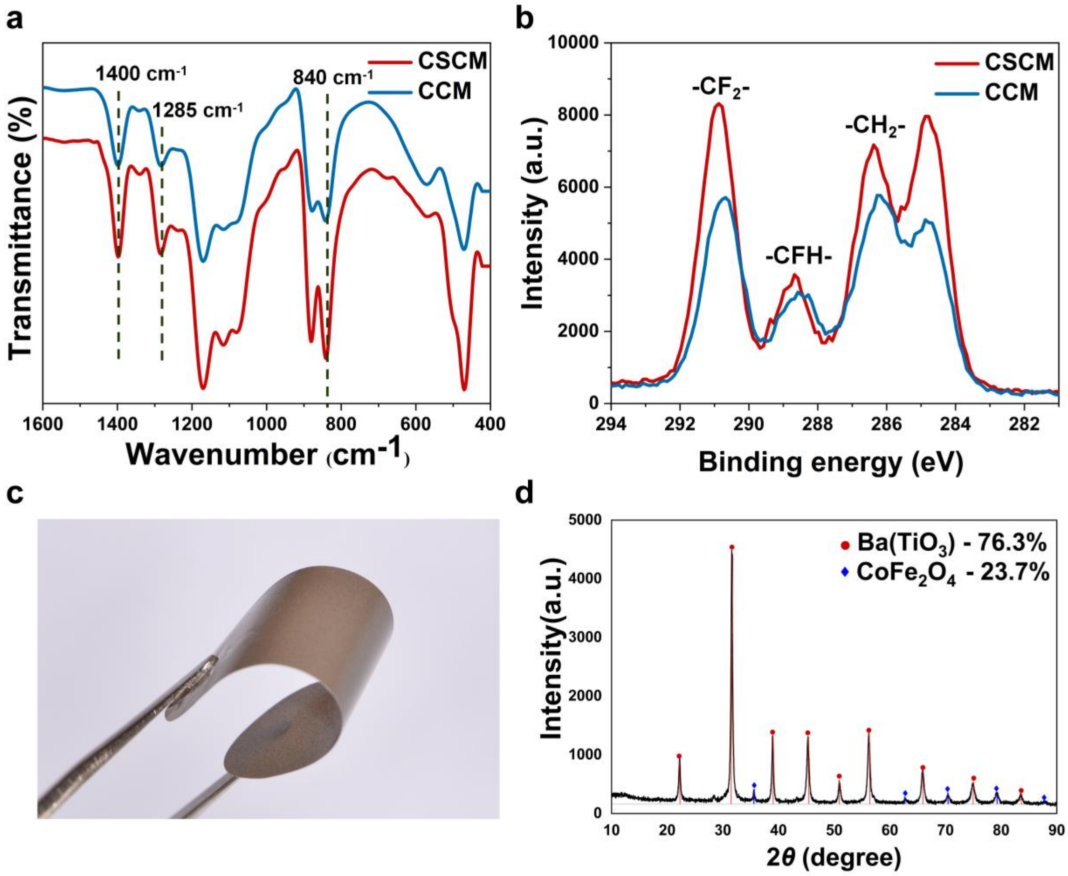

Supplementary Fig. 2 Characterization of the basic properties of composite membranes.

a, FTIR results of CSCM and CCM. **b,** XPS results for CSCM and CCM. (CSCM represents a composite membrane containing 10% CFO@BTO core-shell particles by weight; 2 CSCM means that the weight content of the core-shell particles is 20%, twice that of CSCM; 1/2 CSCM means 5% core-shell particle weight content, which is half that of CSCM). **c,** Image of CFO@BTO/P(VDF-TrFE) membrane (CSCM). **d,** XRD results of CFO@BTO core-shell. Source data are provided as a Source Data file.

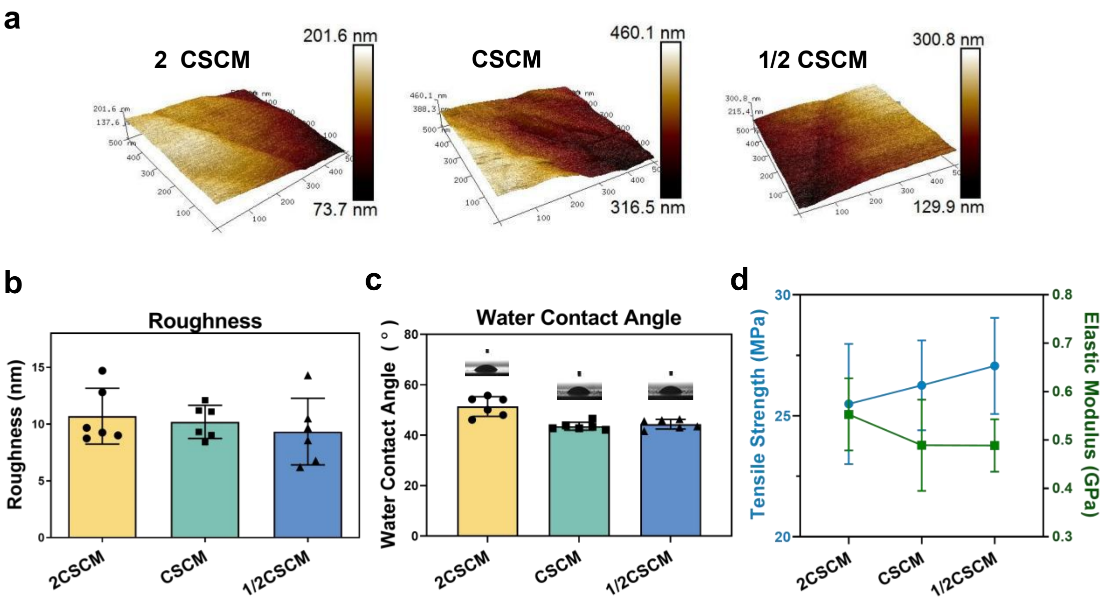

Supplementary Fig. 3 Surface characteristics and mechanical properties of composite membranes.

a, Surface topography and roughness **(b)** measured by AFM of 2 CSCM, CSCM and 1/2 CSCM.There was no difference in surface roughness between the different membrane groups. *n*=6 independent membrane samples; mean±SEM. **c,** Data statistics and photos of water contact angle, which also showed no significant differences. The contact angle values were 80°±, indicating similar hydrophilicity. *n*=6 independent membrane samples; mean±SEM. **d,** Tensile strength and elastic modulus of 2 CSCM, CSCM and 1/2 CSCM. *n*=6 independent membrane samples; mean±SEM. Source data are provided as a Source Data file.

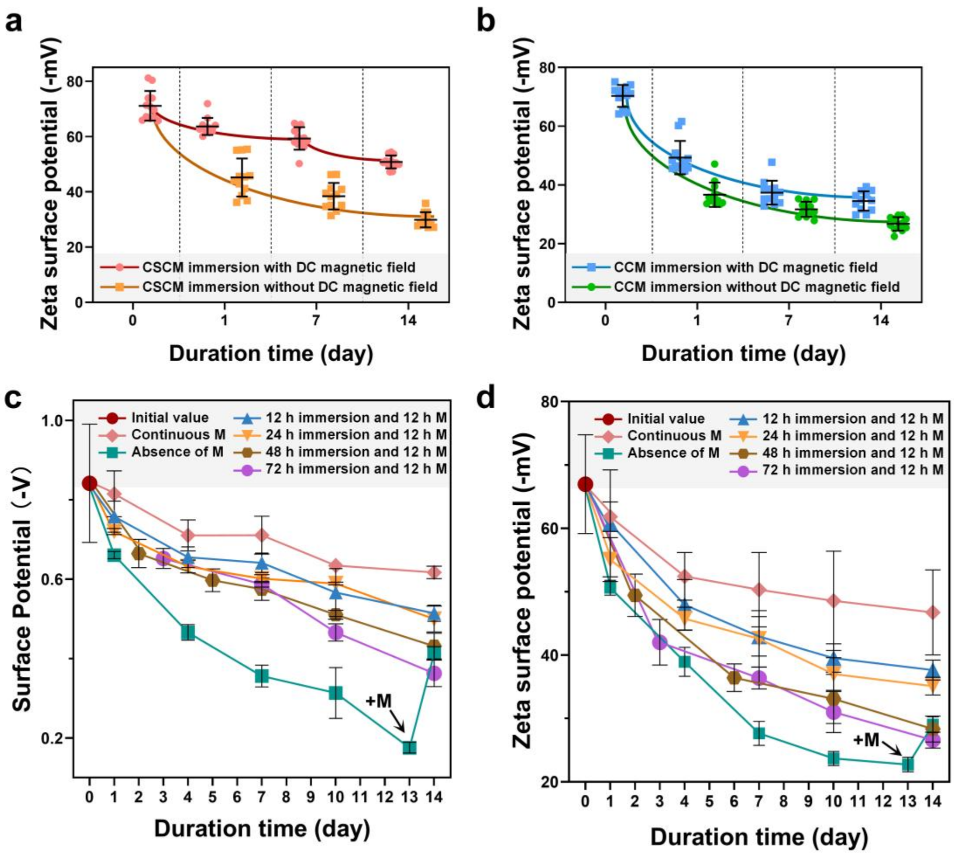

Supplementary Fig. 4 Characterization of the electrical properties of magnetoelectric composite membranes.

a,b, Zeta potential of CSCM **(a)** and CCM **(b)** immersed in phosphate buffer solution (PBS), with or without exposure to a remote DC magnetic field after 1, 7, 14 days. n=12 membranes for per group and per time point; mean±SEM. **c,** Surface potential measured by SKPM and **(d)** zeta potential of CSCM under a continuous magnetic field, 12 h immersion and 12 h magnetic field, 24 h immersion and 12 h magnetic field, 48 h immersion and 12 h magnetic field, 72 h immersion and 12 h magnetic field, and without magnetic field. On the 13th day, a magnetic field was applied to the non-magnetic field exposed group, to observe its reactivation effect on the surface potential. n=6 membranes for per group and per time point; mean±SEM. Source data are provided as a Source Data file.

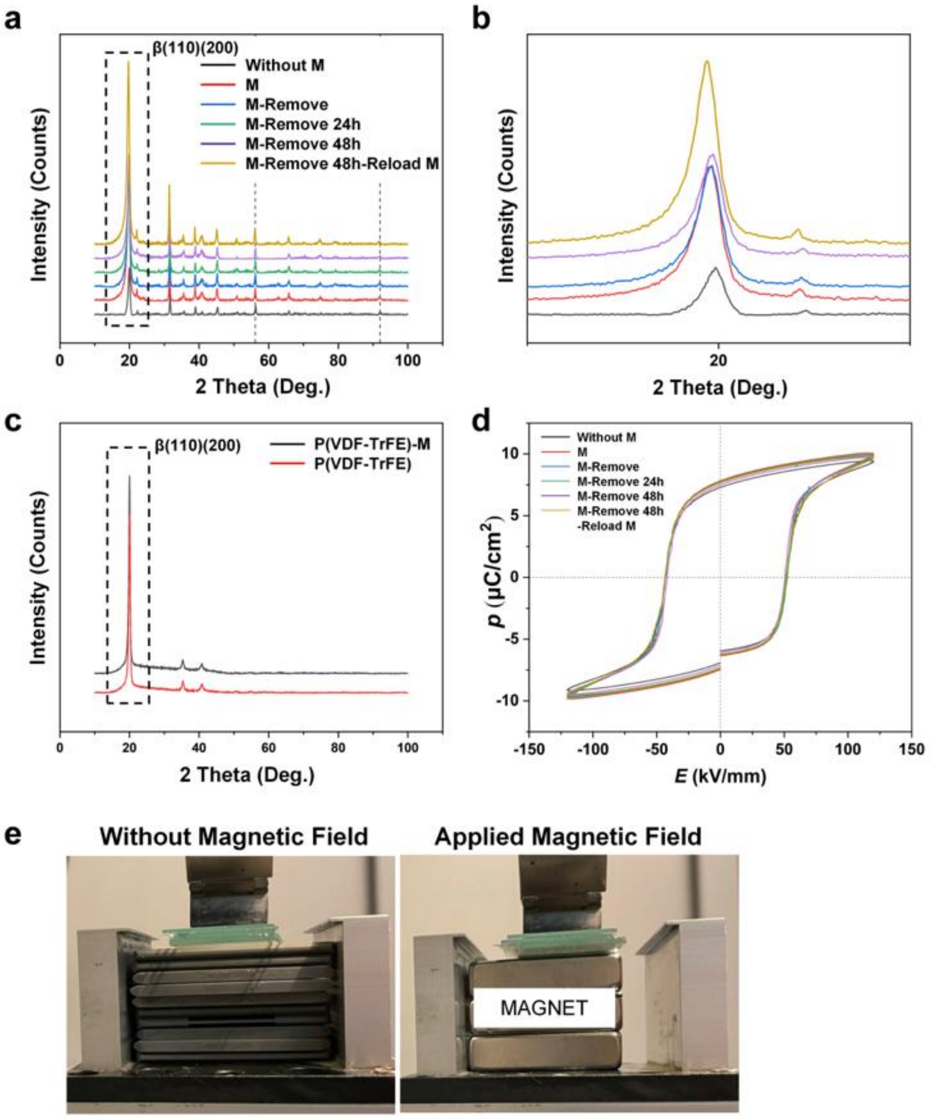

Supplementary Fig. 5 Phase change detection results and device photos.

a, The XRD results of CSCM under different conditions (without magnetic field, applied magnetic field, magnet removal and 24, 48 h after removing magnet and finally reloading magnetic field). **b,** An enlarged view of figure (a), showing the location of the β-phase. According to Bragg Law $\lambda=2d \cdot \sin\theta$, the interplanar crystal spacing of CSCM increased after the magnetic field loading. **c,** The XRD results of P(VDF-TrFE) without magnetic field (black line) and applied magnetic field (red line), which showed no shift of the β-phase position. **d,** The electric hysteresis loop of CSCM under different conditions. **e,** Pictures of the device for XRD detection. Source data are provided as a Source Data file.

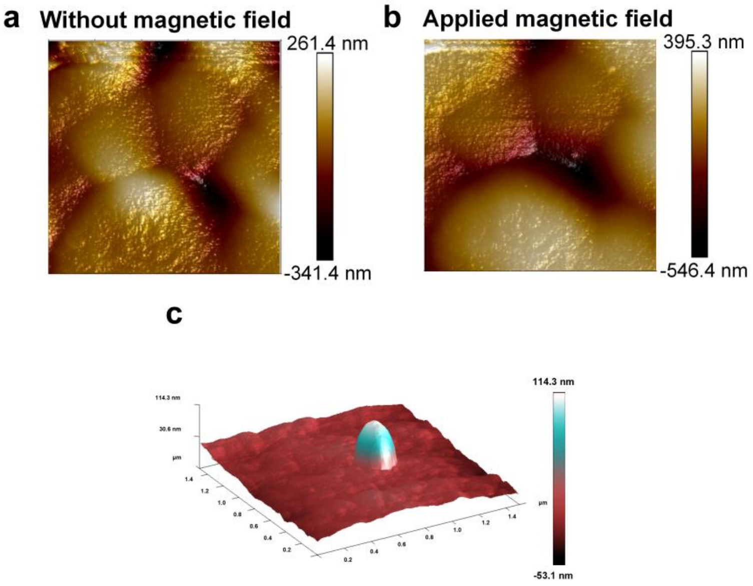

Supplementary Fig. 6 Morphologies of surface potential and interfacial polarization detection

a, The topography shows the instant change of surface potential before and after applying magnetic field *in situ* **(b)**. **c,** 3D topography image of detecting the interface polarization.

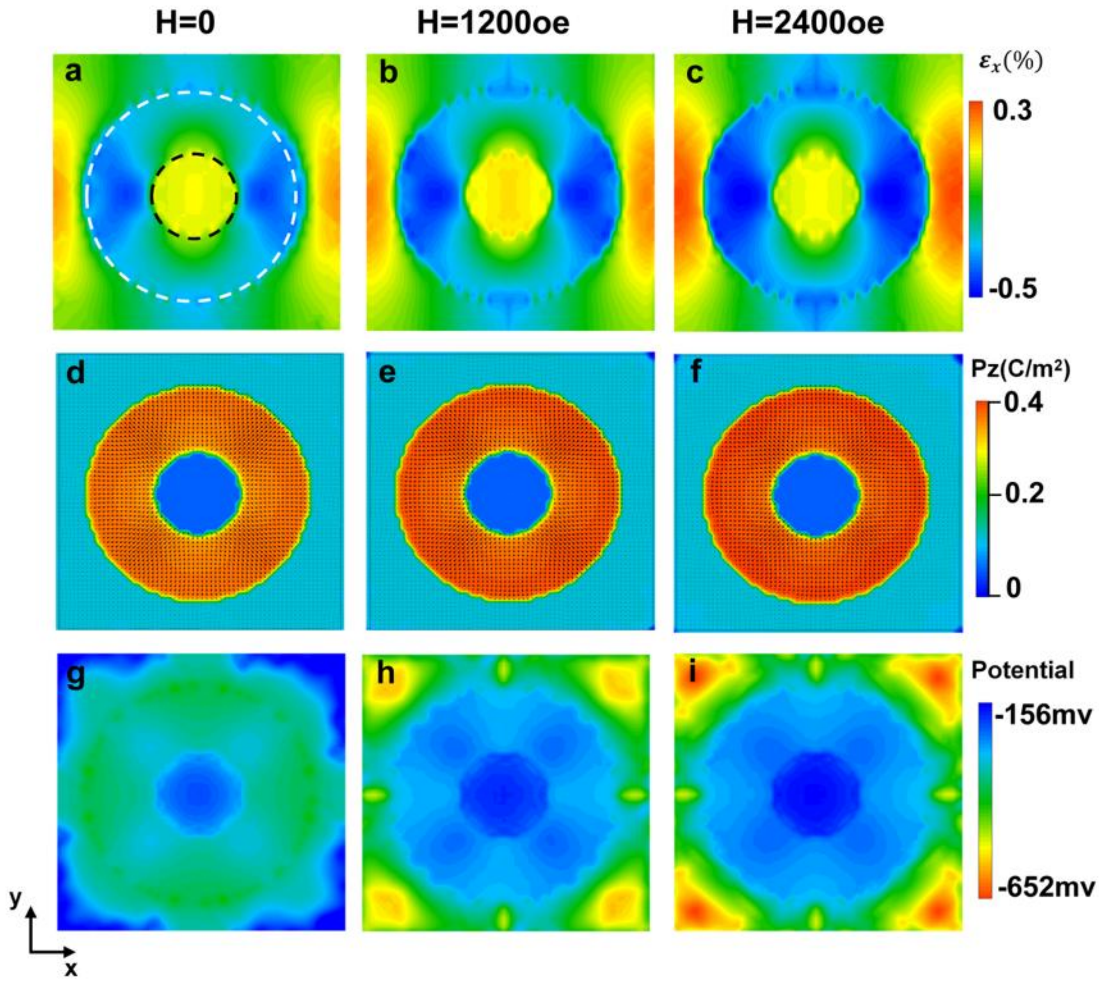

Supplementary Fig. 7 Phase-field simulation of the strain distributions along the x-direction under different magnetic fields **(a)** H=0 **(b)** H=1200 Oe **(c)** H=2400 Oe. The polarization component P_z distributions under different magnetic fields **(d)** H=0 **(e)** H=1200 Oe **(f)** H=2400 Oe. The electrical potential distributions under different magnetic fields **(g)** H=0 **(h)** H=1200 Oe **(i)** H=2400 Oe.

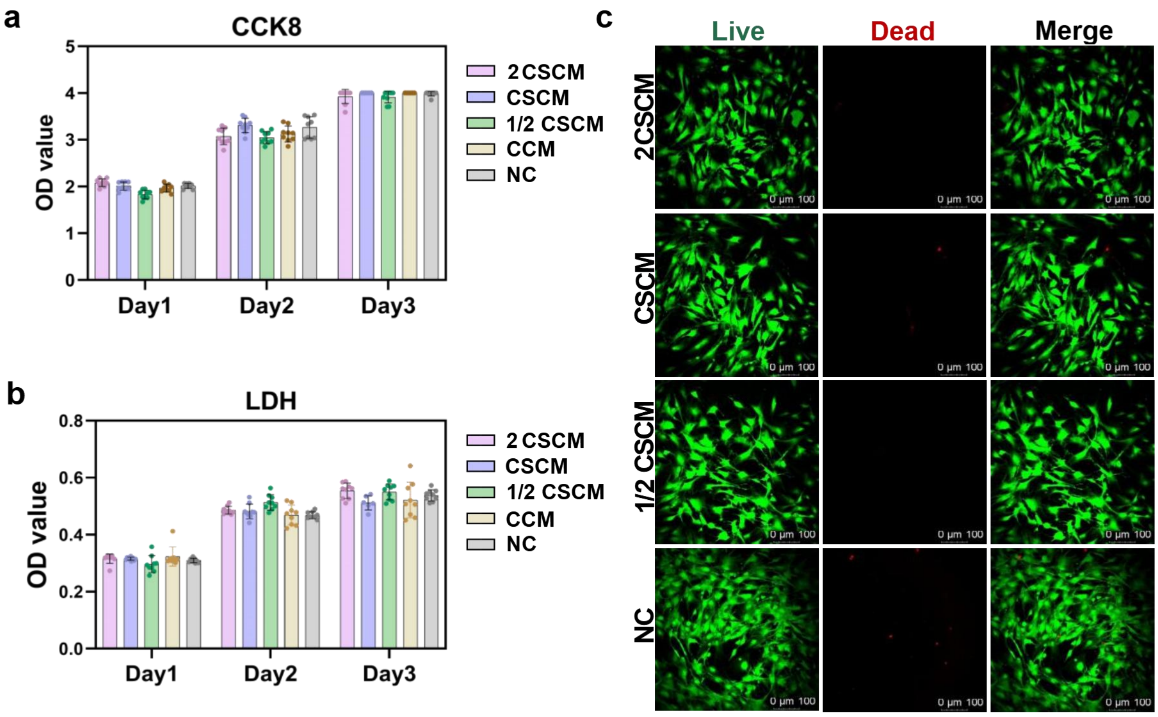

Supplementary Fig. 8 Biocompatibility of composite membranes in the various groups.

a, Cell proliferation of BM-MSCs cultured on the membranes of various groups by the CCK-8 assay after 1, 2 and 3 days. *n*=9 biologically independent samples; mean±SEM; one-way ANOVA. **b,** The lactate dehydrogenase content in the supernatants of BM-MSCs cultured on the membranes after 1, 2 and 3 days. **c,** Live and dead cell staining on the membranes of different groups. *n*=9 biologically independent samples; mean±SEM; one-way ANOVA. Source data are provided as a Source Data file.

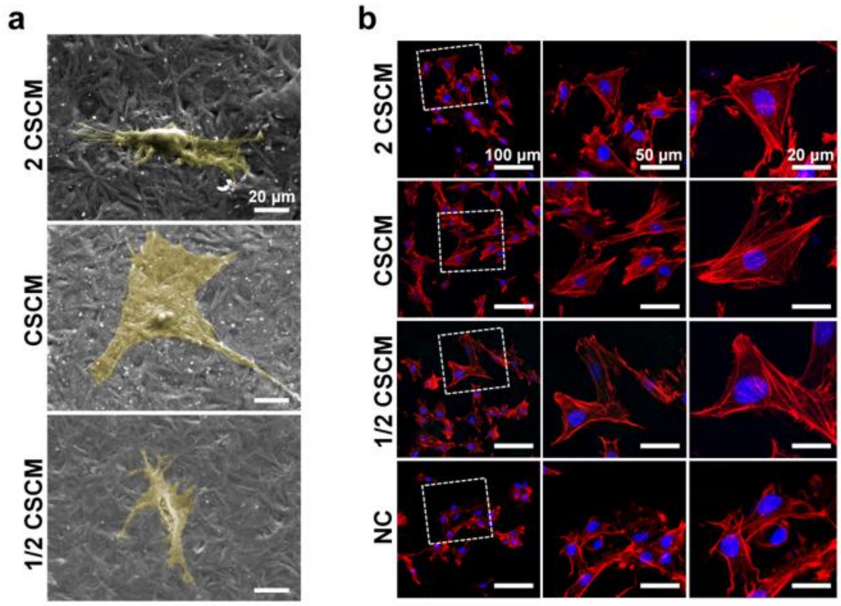

Supplementary Fig. 9 Characterization of cell spreading on the membrane surfaces.

a, SEM and immunofluorescence images **(b)** of cell spreading on the different membrane groups. At least three times of experiments were repeated independently.

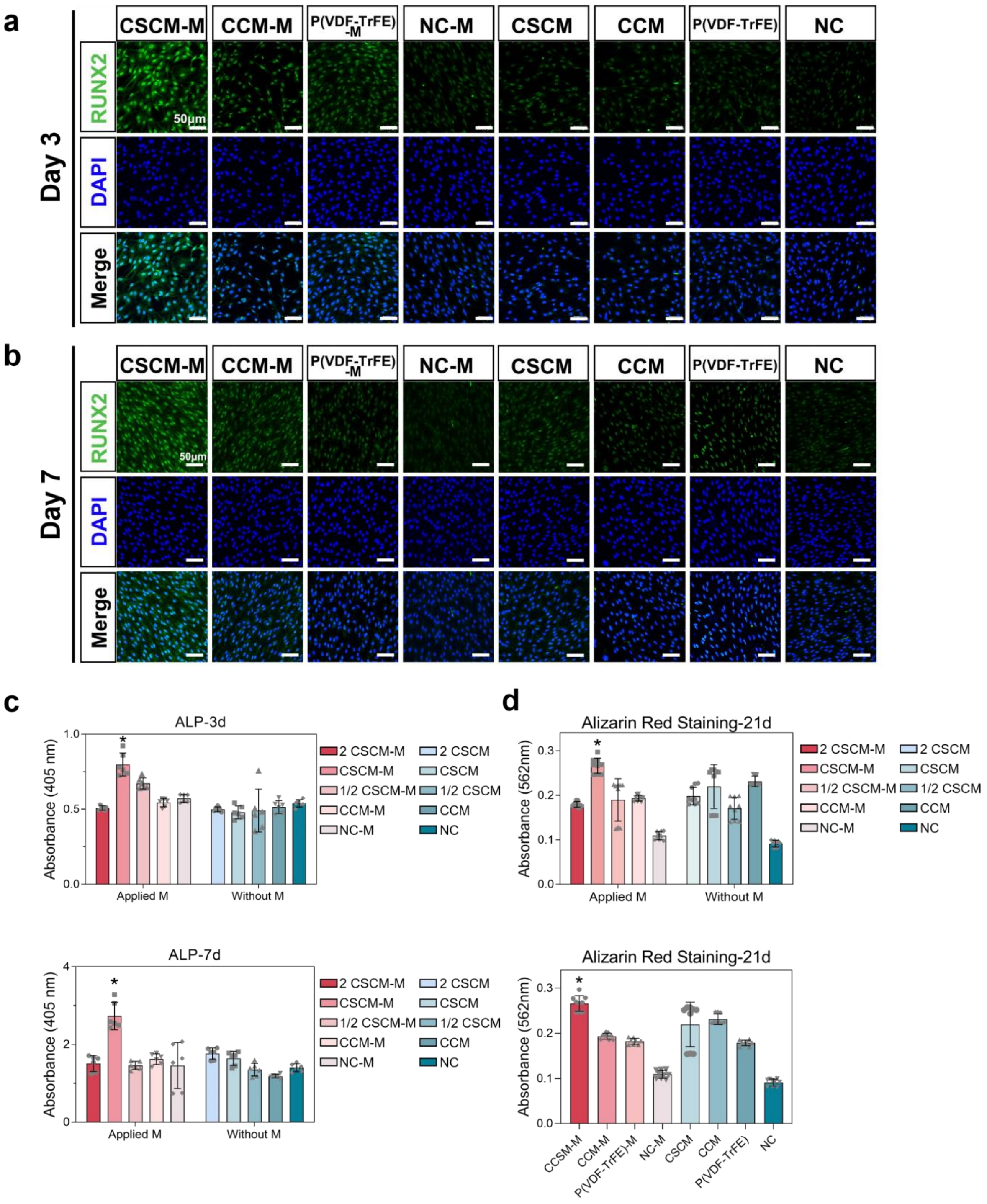

Supplementary Fig. 10 Osteogenic properties of core-shell magnetoelectric composite membranes

a,b, Immunofluorescence images showed the upregulated expression of RUNX2 in the CSCM-M group after 3 and 7 days of cell culture (Scale bars: 50 μm). **c,** ALP activity after 3 and 7 days of cell culture. *n*=9 biologically independent samples; mean±SEM. *P<0.05, one-way ANOVA. **d,** Quantification of Alizarin red staining after 21 days of cell culture. *n*=9 biologically independent samples; mean±SEM. *P<0.05, one-way ANOVA. Source data are provided as a Source Data file.

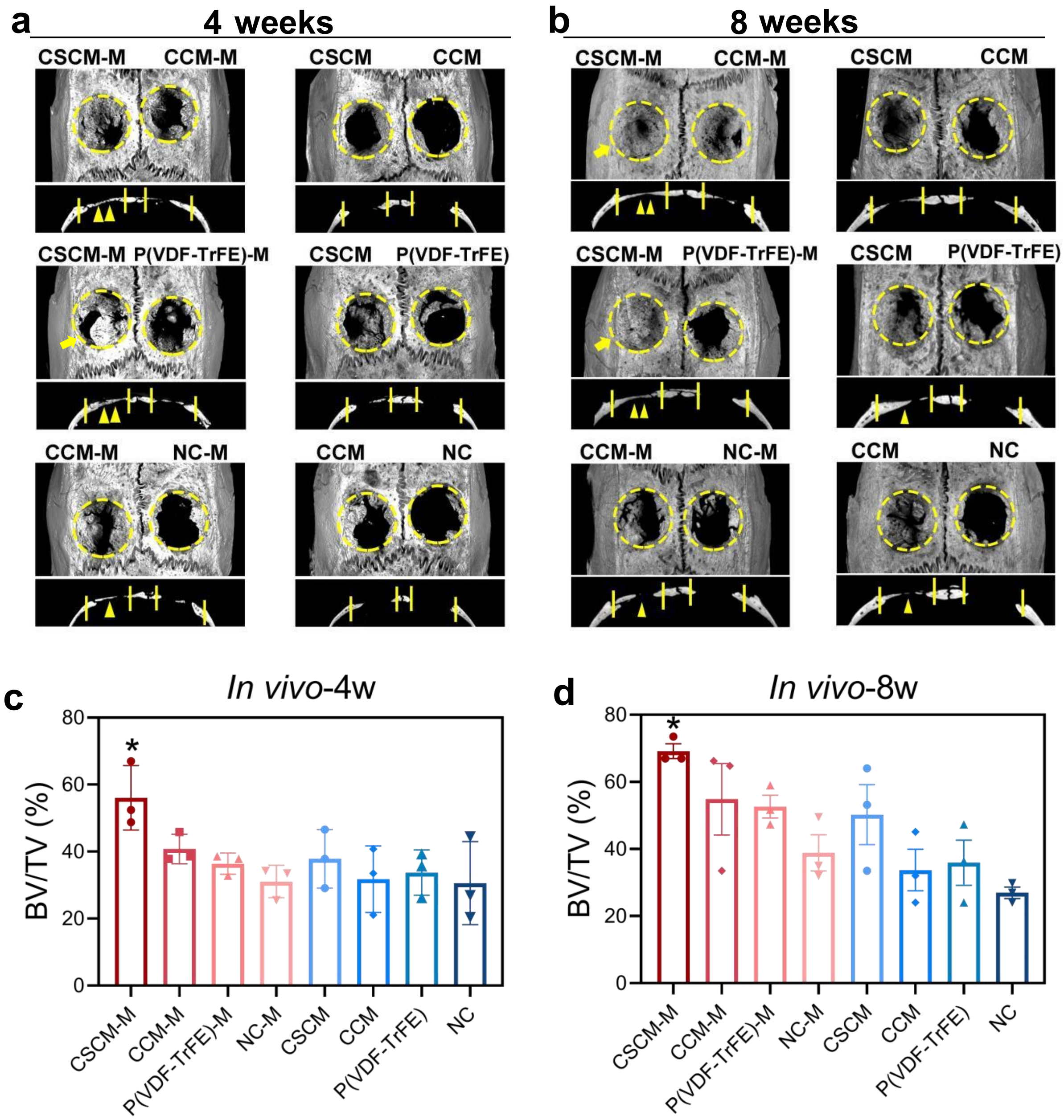

Supplementary Fig. 11 | Osteogenic properties of core-shell magnetoelectric composite membranes *in vivo*.

a,b, Micro-CT images of bone regeneration in rat cranial defects at 4 **(a)** and 8 **(b)** weeks after membrane implantation, showing the most abundant new bone formation in the CSCM-M group. (Yellow arrows indicated enhanced bone regeneration in the CSCM-M group. Yellow triangles denote the new bone. Yellow dotted lines denote the boundary between nascent bone and host bone.) **c,d,** Quantitative statistics of new bone from Micro-CT analysis. n=3 rats for per group and per time point; mean±SEM. *P<0.05, one-way ANOVA. Source data are provided as a Source Data file.

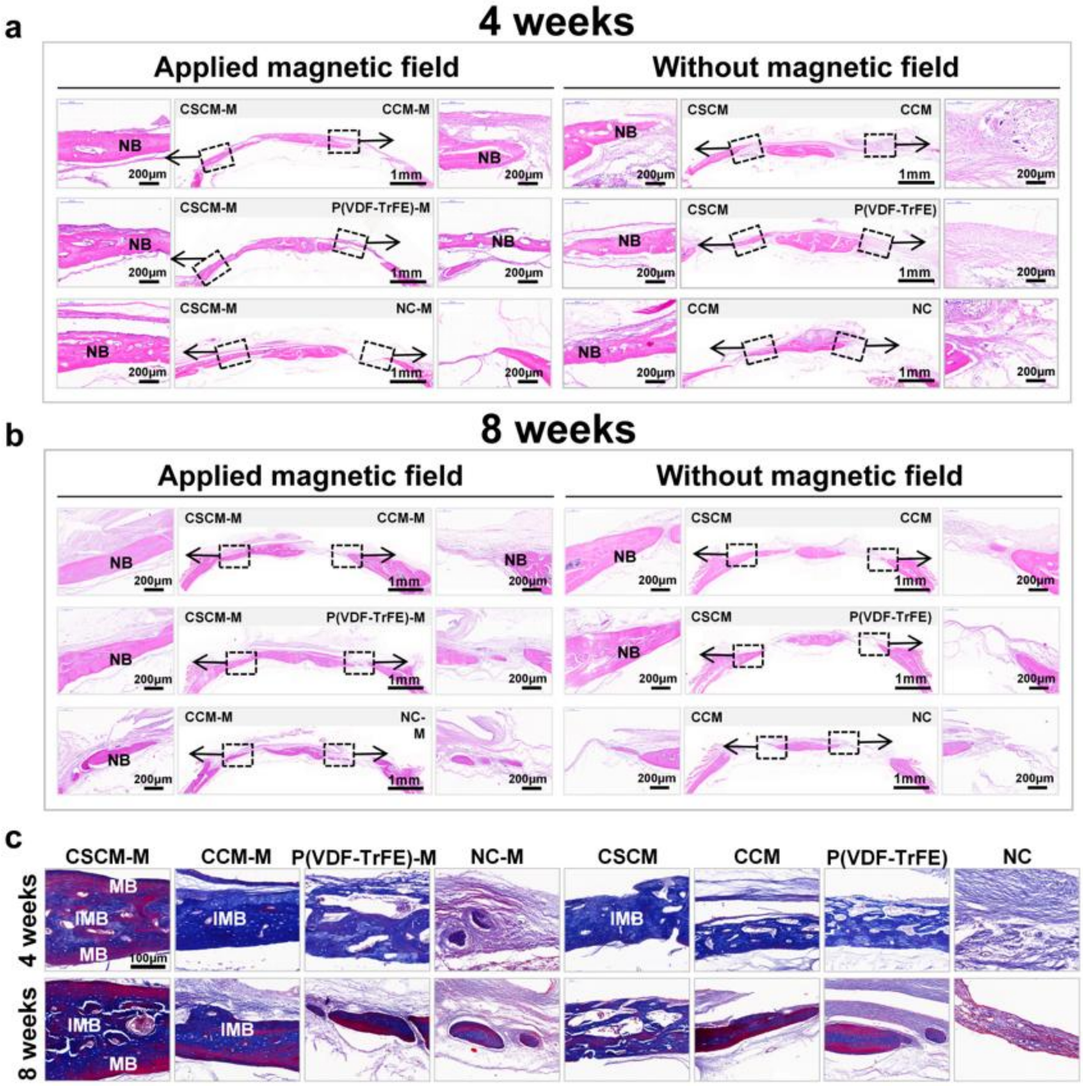

Supplementary Fig. 12 | Osteogenic properties of core-shell magnetoelectric composite membranes *in vivo*

a,b, H&E staining (NB: nascent bone) at 4, 8 weeks after implantation. **c,** Masson's trichrome staining (MB: mature bone; IMB: immature bone). At least three times of experiments were repeated independently.

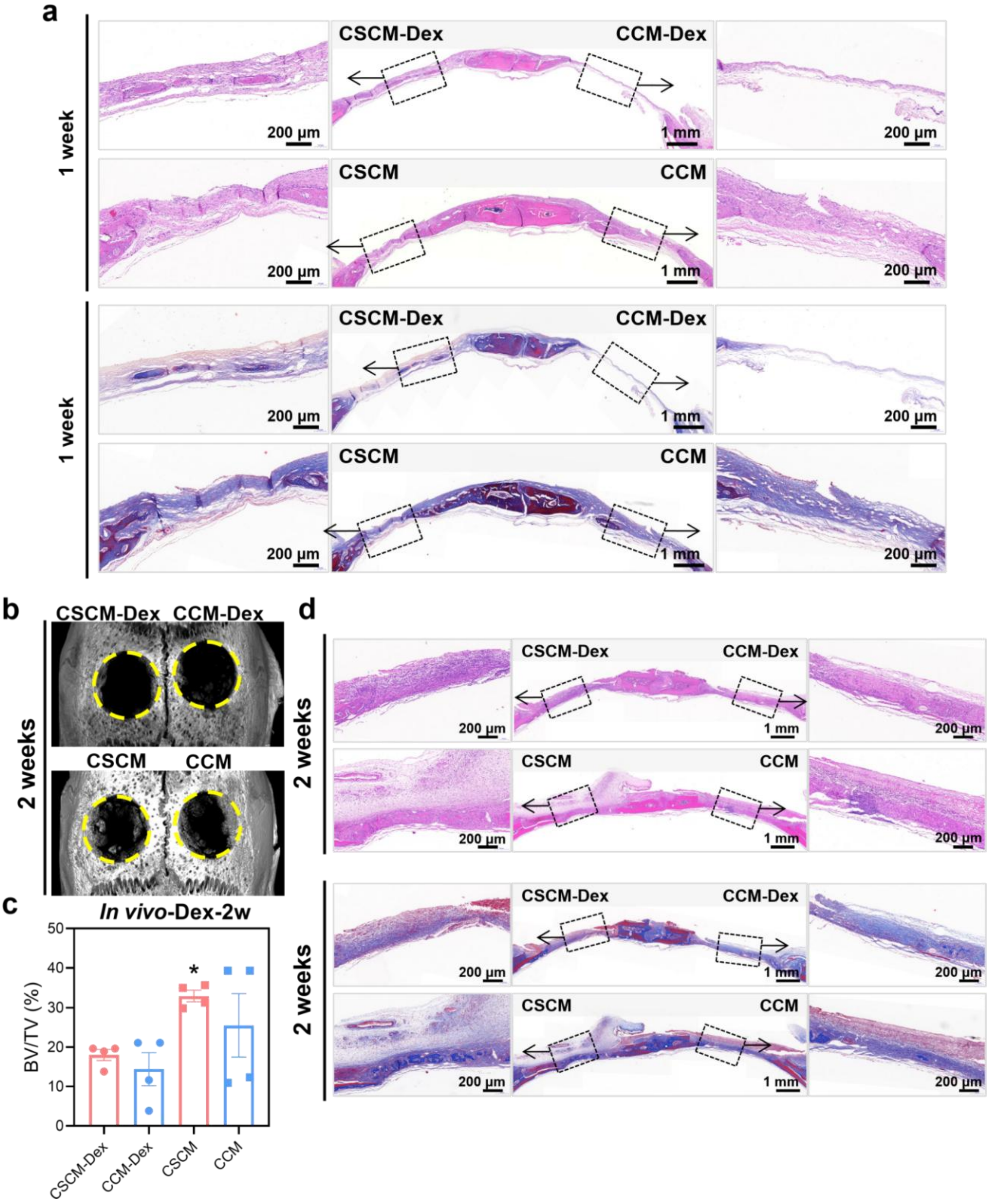

Supplementary Fig. 13 | Osteogenic inhibition by high dose dexamethasone *in vivo*.

a, H&E staining and Masson's trichrome staining at 1 week, after 3 days implantation and dexamethasone injection. **b,** Micro-CT images of bone regeneration in rat cranial defects at 2 weeks after 3 days of membrane implantation and dexamethasone injection. **c,** Quantitative statistics of new bone from micro-CT analysis. n=4 rats for per group and per time point; mean±SEM.*$P<0.05$, one-way ANOVA. **d,** H&E staining and Masson's trichrome staining at 1 week after 3 days implantation and dexamethasone injection. At least three times of experiments were repeated independently. Source data are provided as a Source Data file.

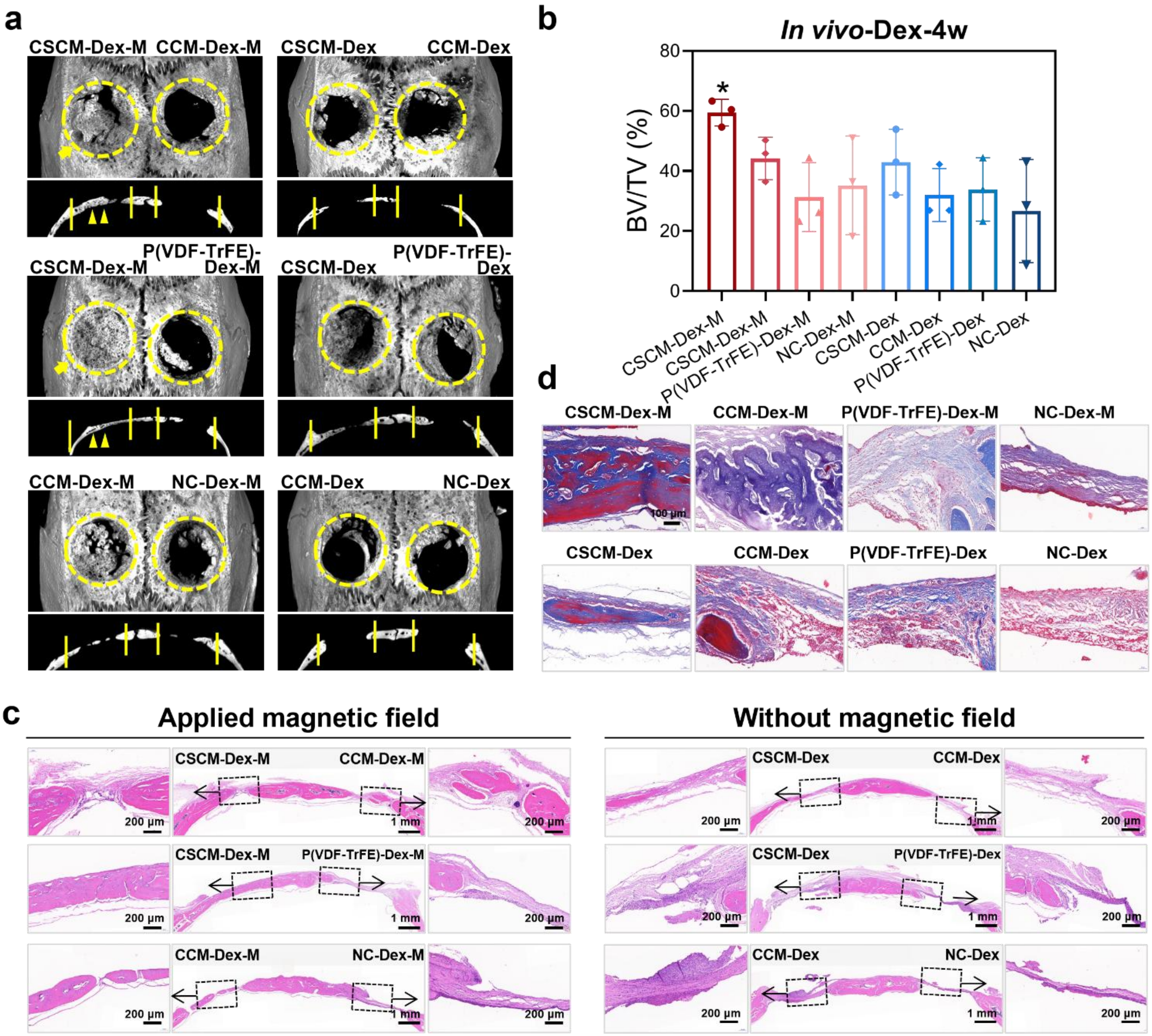

Supplementary Fig. 14 | The reactivation effect of the implanted core-shell magnetoelectric composite membrane *in vivo* within the rat skull defect model with osteogenesis inhibition.

a, Micro CT images and quantitative statistics **(b)** of bone volume after dexamethasone (Dex) injection to inhibit osteogenesis with and without magnetic treatment (4 weeks). *n*=3 rats for per group; mean±SEM. *P<0.05, one-way ANOVA.

c, H&E staining and Masson's trichrome staining **(d)** of new bone formation after dexamethasone (Dex) injection to inhibit osteogenesis with and without magnetic treatment (4 weeks). Source data are provided as a Source Data file. At least three times of experiments were repeated independently.

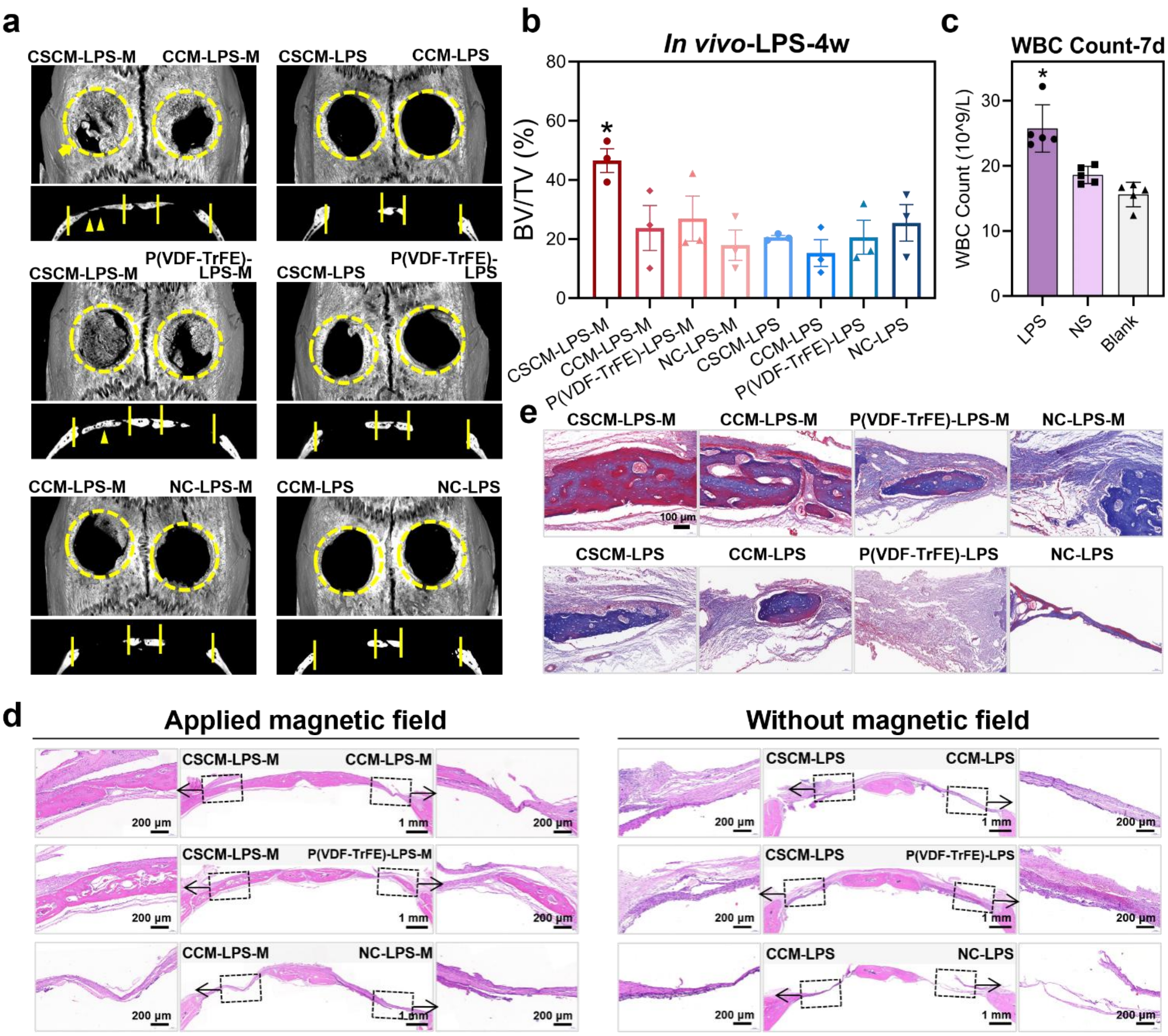

Supplementary Fig. 15 | The reactivation effect of implanted core-shell magnetoelectric composite membrane *in vivo* within the rat skull defect model with LPS-induced systemic inflammation.

a, Micro CT images and quantitative statistics **(b)** of bone volume after LPS injection to induce systemic inflammation with and without magnetic treatment (4 weeks). *n*=3 rats for per group; mean±SEM. *P<0.05, one-way ANOVA. **c,** White blood cell count at 7 d after membrane implantation. (LPS-1 mg/kg LPS injection, NS-normal saline injection, Blank-without any injection). *n*=5 rats for per group; mean±SEM. *P<0.05, one-way ANOVA. **d,** H&E staining and Masson's trichrome staining **(e)** of new bone formation after LPS injection to induce systemic inflammation with and without magnetic treatment (4 weeks). Source data are provided as a Source Data file. At least three times of experiments were repeated independently.

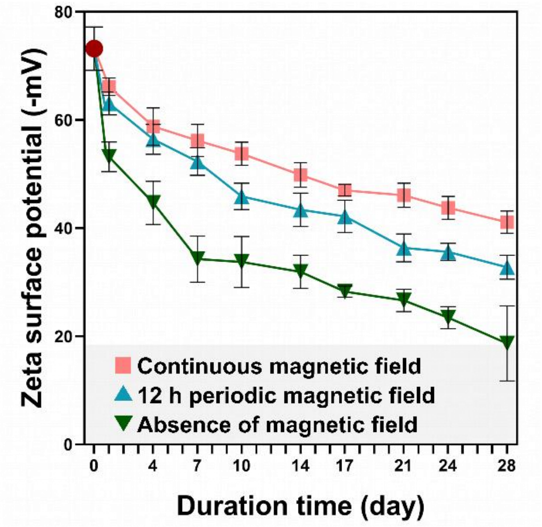

Supplementary Fig. 16 The surface potentials of the membranes immersed in culture medium decreased over time. *n*=6 independent membrane samples for per group and per time point; mean±SEM. Source data are provided as a Source Data file.

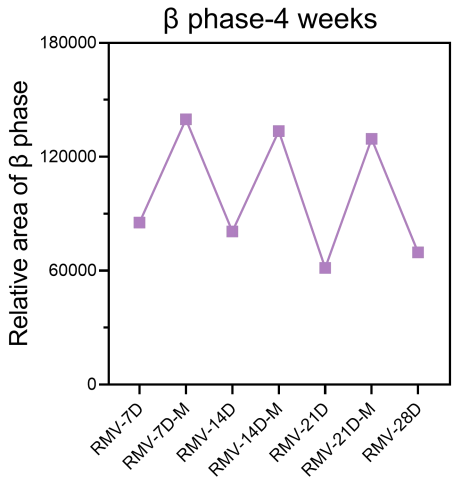

Supplementary Fig. 17 Relative area of β-phase calculated from XRD. The β-phase transition was detected from day 7 to day 28 after the magnetic field was removed and 3 cycles were performed. Source data are provided as a Source Data file.

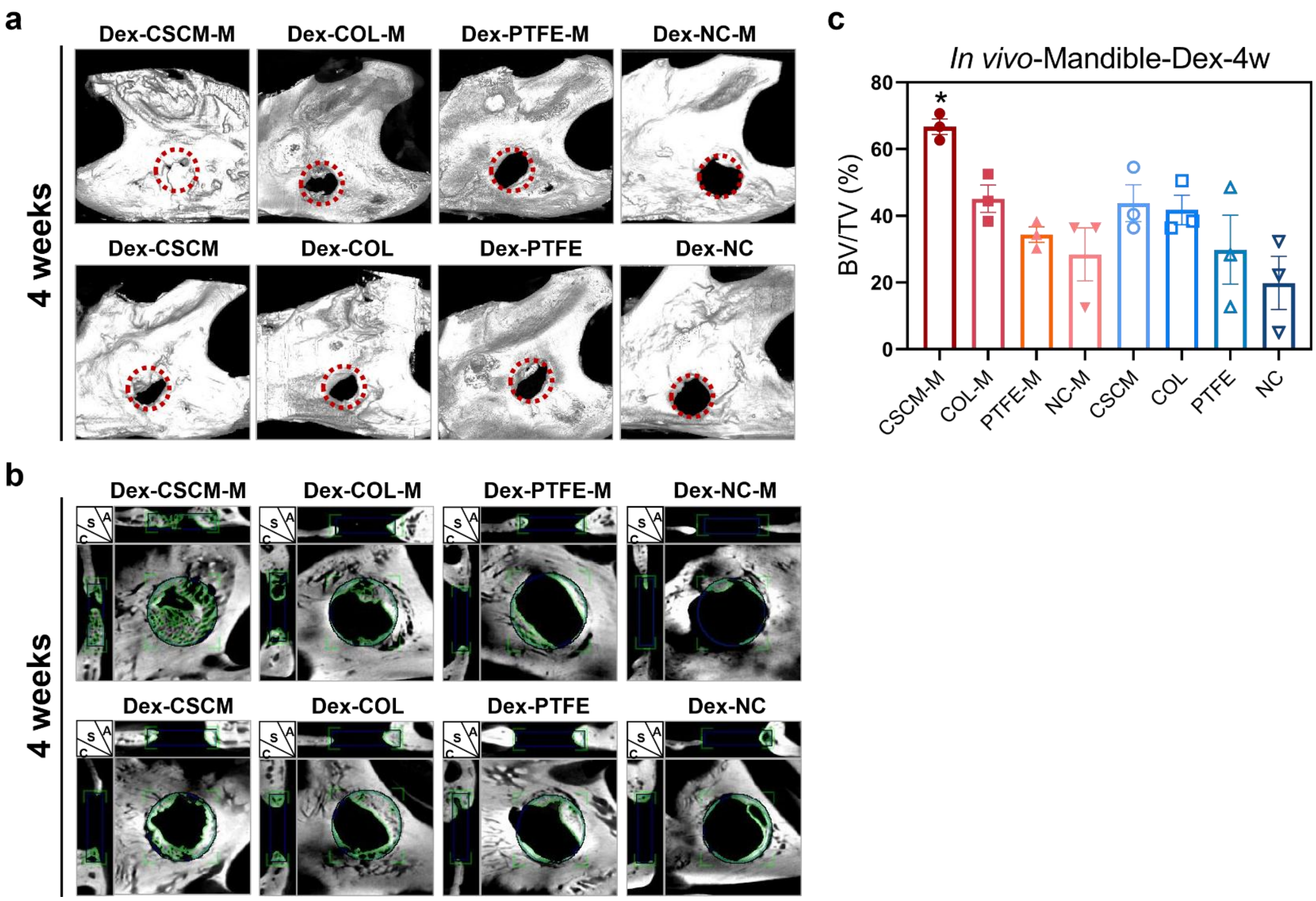

Supplementary Fig. 18 Comparison of osteogenic effects of CSCM versus commercially-available membranes in promoting bone defect repair. The model of mandibular critical defect (3 mm) combined with Dex-induced osteogenic inhibition in rats were used in this study. **a,** Three-dimensional micro-CT images at four weeks after material implantation, with and without magnetic field treatment (4 weeks). The bone defect areas are marked by a red dotted circle. **b,** Two-dimensional images of the defect areas at 4 weeks after material implantation and co-morbidity model preparation. The bone defect areas are marked by circles or rectangles. S=Sagittal plane, C=Coronal plane, A=Cross section. **c,** Quantitative statistics of the ratio of new bone volume to total volume (BV/TV). *n*=3 rats for per group; mean±SEM. *P<0.05, one-way ANOVA. Source data are provided as a Source Data file.

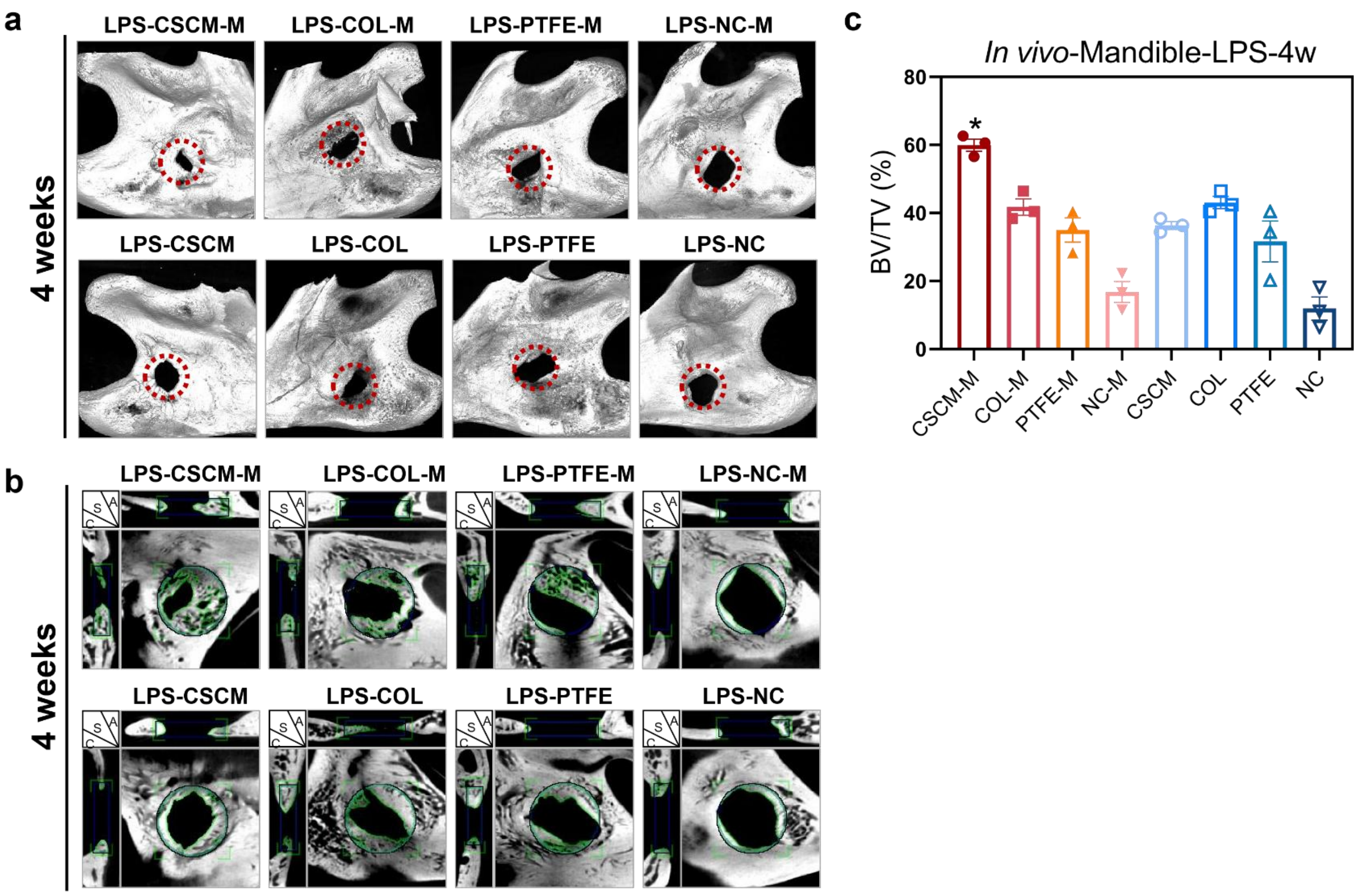

Supplementary Fig.19 Comparison of osteogenic effects of CSCM versus commercially-available membranes in promoting bone defect repair. The model of mandibular 3 mm critical defect combined LPS-induced systemic inflammation in rats were used in this study. **a,** Three-dimensional micro-CT images of four weeks after material implantation, with and without magnetic field treatment (4 weeks). The bone defect areas were marked by a red dotted circle. **b,** Two-dimensional images of the defect areas 4 weeks after material implantation and comorbid model preparation. The bone defect areas were marked by circles or rectangles. S=Sagittal plane, C=Coronal plane, A=Cross section. **c,** Quantitative statistics of the ratio of new bone volume to total volume (BV/TV). *n*=3 rats for per group; mean±SEM. *P<0.05, one-way ANOVA. Source data are provided as a Source Data file.

Table S1 Osteogenesis efficiency and material morphology of the referenced materials

Material name	Morphology	Stimulus type	Data form	Osteogenesis efficiency (%)	References
BTO NP /P(VDF-TrFE)	membrane	Electroactive	BV/TV	56 (12 w); 50 (4 w)	(1)
BFO+	membrane	Electroactive	BV/TV	54 (8 w)	(2)
P(VDF-TrFE)	membrane	Electroactive	BV/TV	52 (8 w)	(3)
KNN	ceramic	Electroactive	BV	1.2 mm^3	(4)
AGCP	gel	Electroactive	BV/TV	53 (12 w)	(5)
Actuator	scaffold	Electroactive	Area	40 (4 w)	(6)
PVDF-TrFE/BT	scaffold	Electroactive	BV/TV	20 (4 w)	(7)
GO-CMC	scaffold	Electroactive	BV/TV	30±	(8)
nHA/GO/CS+	scaffold	Photothermal	BV/TV	20 (8 w)	(9)
5BCN@AKT	scaffold	Photothermal	BV/TV	58.2 (8 w)	(10)
$GdPO_4$/CS/Fe_3O_4	scaffold	Photothermal	BV/TV	61.23 (12w)	(11)
TCP-PDLLA-5LB	scaffold	Photothermal	Area	35 (8 w)	(12)
BP-$SrCl_2$/PLGA +Laser	microsphere	Photothermal	BV/TV	38 (8 w)	(13)
BPs@PLGA+NIR	membrane	Photothermal	BV/TV	29.57 (10 w)	(14)
ACS+NSD-BMP+ US	liposome	Sonodynamic	BV/TV	20 (4 w)	(15)
LIPUS+$BaTiO_3$/pTi	scaffold	Sonodynamic	BV/TV	30 (12 w)	(16)
α-TCP/CS/Fe_3O_4/GO	cement	Magnetic	BV/TV	18 (8 w)	(17)
AKT-Fe_3O_4-CaO_2	scaffold	Magnetic	BV/TV	12 (8 w)	(18)
BMSC-Fe_3O_4-SMF-Exos	particles	Magnetic	BV/TV	49 (12 w)	(19)
Magnetic scaffold B	scaffold	Magnetic	Area	36.23 (8 w)	(20)
Iron oxide core coated with PEI-B	particles	Magnetic	Healing	43 (8 w)	(21)
CS/Col/Fe_3O_4 /nHAP	scaffold	Magnetic	BV/TV	23 (8 w)	(22)
CFO/P(VDF-TrFE) -E/M	membrane	Magneto-electric	BV/TV	52.41 (8 w)	(23)
CFO@BTO/P(VDF-TrFE)-M	membrane	Magneto-electric	BV/TV	69.33 (8 w)	Our work

Table S1. Studies using animal experiments to assess *in vivo* osteogenesis efficiency were included.

Based on a natural bone healing time of 12 weeks, this table only included *in vivo* studies that lasted no longer than 12 weeks to compare the efficiency of biomaterials. All included biomaterials and the results of CSCM-M under both conventional and co-morbidity conditions were concluded in Fig 1a.

Table S2 Osteogenesis efficiency (%) at 4 weeks post-implantation

Group Name	Bone defect	Bone defect with Co-morbidity
CSCM-M	56.05	52.08
CCM-M	40.76	33.67
CSCM	37.85	29.63
CCM	31.78	22.99

Supplementary References

1 Zhang, X. *et al.* Nanocomposite Membranes Enhance Bone Regeneration Through Restoring Physiological Electric Microenvironment. *ACS Nano* **10**, 7279-7286, doi:10.1021/acsnano.6b02247 (2016).

2 Liu, Y. *et al.* Built-In Electric Fields Dramatically Induce Enhancement of Osseointegration. *Advanced Functional Materials* **27**, doi:10.1002/adfm.201703771 (2017).

3 Zhang, C. *et al.* Modulating Surface Potential by Controlling the beta Phase Content in Poly(vinylidene fluoridetrifluoroethylene) Membranes Enhances Bone Regeneration. *Adv Healthc Mater* **7**, e1701466, doi:10.1002/adhm.201701466 (2018).

4 Yu, P. *et al.* Bone-Inspired Spatially Specific Piezoelectricity Induces Bone Regeneration. *Theranostics* **7**, 3387-3397, doi:10.7150/thno.19748 (2017).

5 Kapat, K. *et al.* Osteochondral Defects Healing Using Extracellular Matrix Mimetic Phosphate/Sulfate Decorated GAGs-Agarose Gel and Quantitative Micro-CT Evaluation. *ACS Biomater Sci Eng* **5**, 149-164, doi:10.1021/acsbiomaterials.8b00253 (2019).

6 Reis, J. *et al.* A new piezoelectric actuator induces bone formation *in vivo*: a preliminary study. *J Biomed Biotechnol* **2012**, 613403, doi:10.1155/2012/613403 (2012).

7 Freitas, G. P. *et al.* Potential of Osteoblastic Cells Derived from Bone Marrow and Adipose Tissue Associated with a Polymer/Ceramic Composite to Repair Bone Tissue. *Calcif Tissue Int* **101**, 312-320, doi:10.1007/s00223-017-0282-3 (2017).

8 Ruan, J. *et al.* Enhanced Physiochemical and Mechanical Performance of Chitosan-Grafted Graphene Oxide for Superior Osteoinductivity. *Advanced Functional Materials* **26**, 1085-1097, doi:10.1002/adfm.201504141 (2016).

9 Ma, L. *et al.* A novel photothermally controlled multifunctional scaffold for clinical treatment of osteosarcoma and tissue regeneration. *Materials Today* **36**, 48-62, doi:10.1016/j.mattod.2019.12.005 (2020).

10 Zhao, C., Shen, A., Zhang, L., Lin, K. & Wang, X. Borocarbonitrides nanosheets engineered 3D-printed scaffolds for integrated strategy of osteosarcoma therapy and bone regeneration. *Chemical Engineering Journal* **401**, doi:10.1016/j.cej.2020.125989 (2020).

11 Zhao, P.-P. *et al.* Ordered arrangement of hydrated $GdPO_4$ nanorods in magnetic chitosan matrix promotes tumor photothermal therapy and bone regeneration against breast cancer bone metastases. *Chemical Engineering Journal* **381**, doi:10.1016/j.cej.2019.122694 (2020).

12 Dang, W. *et al.* LaB6 surface chemistry-reinforced scaffolds for treating bone tumors and bone defects. *Applied Materials Today* **16**, 42-55, doi:10.1016/j.apmt.2019.04.015 (2019).

13 Wang, X. *et al.* Near-infrared light-triggered drug delivery system based on black phosphorus for *in vivo* bone regeneration. *Biomaterials* **179**, 164-174, doi:10.1016/j.biomaterials.2018.06.039 (2018).

14 Tong, L. *et al.* Near-infrared light control of bone regeneration with biodegradable photothermal osteoimplant. *Biomaterials* **193**, 1-11, doi:10.1016/j.biomaterials.2018.12.008 (2019).

15 Crasto, G. J. *et al.* Controlled bone formation using ultrasound-triggered release of BMP-2 from liposomes. *J. Control. Release* **243**, 99-108, doi:10.1016/j.jconrel.2016.09.032 (2016).

16 Fan, B. *et al.* Electroactive barium titanate coated titanium scaffold improves osteogenesis and osseointegration with low-intensity pulsed ultrasound for large segmental bone defects.

Bioactive materials **5**, 1087-1101, doi:10.1016/j.bioactmat.2020.07.001 (2020).

17 Yan, F. *et al.* Biphasic Injectable Bone Cement with Fe_3O_4/GO Nanocomposites for the Minimally Invasive Treatment of Tumor-Induced Bone Destruction. *ACS Biomater Sci Eng* **5**, 5833-5843, doi:10.1021/acsbiomaterials.9b00472 (2019).

18 Dong, S., Chen, Y., Yu, L., Lin, K. & Wang, X. Magnetic Hyperthermia–Synergistic H_2O_2 Self-Sufficient Catalytic Suppression of Osteosarcoma with Enhanced Bone-Regeneration Bioactivity by 3D-Printing Composite Scaffolds. *Advanced Functional Materials* **30**, doi:10.1002/adfm.201907071 (2019).

19 Wu, D. *et al.* Bone mesenchymal stem cells stimulation by magnetic nanoparticles and a static magnetic field: release of exosomal miR-1260a improves osteogenesis and angiogenesis. *J Nanobiotechnology* **19**, 209, doi:10.1186/s12951-021-00958-6 (2021).

20 Panseri, S. *et al.* Modifying bone scaffold architecture *in vivo* with permanent magnets to facilitate fixation of magnetic scaffolds. *Bone* **56**, 432-439, doi:10.1016/j.bone.2013.07.015 (2013).

21 Brett, E. *et al.* Magnetic Nanoparticle-Based Upregulation of B-Cell Lymphoma 2 Enhances Bone Regeneration. *Stem Cells Transl Med* **6**, 151-160, doi:10.5966/sctm.2016-0051 (2017).

22 Zhao, Y. *et al.* Magnetic bioinspired micro/nanostructured composite scaffold for bone regeneration. *Colloids Surf B Biointerfaces* **174**, 70-79, doi:10.1016/j.colsurfb.2018.11.003 (2019).

23 Liu, W. *et al.* Remote Tuning of Built‐In Magnetoelectric Microenvironment to Promote Bone Regeneration by Modulating Cellular Exposure to Arginylglycylaspartic Acid Peptide. *Advanced Functional Materials* **31**, 2006226, doi:10.1002/adfm.202006226 (2020).
