# Supplementary material for: In situ activation of flexible magnetoelectric membrane enhances bone defect repair
Source: Nat Commun. 2023 Jul 10;14:4091. doi: 10.1038/s41467-023-39744-3 (PMC10333320; doi:10.1038/s41467-023-39744-3)
Supplement: Supplementary file 3 — Reporting Summary [file 41467_2023_39744_MOESM3_ESM.pdf]

nature portfolio

Corresponding author(s): Xuliang Deng

Last updated by author(s): May 25, 2023

Reporting Summary

Nature Portfolio wishes to improve the reproducibility of the work that we publish. This form provides structure for consistency and transparency in reporting. For further information on Nature Portfolio policies, see our Editorial Policies and the Editorial Policy Checklist.

Statistics

For all statistical analyses, confirm that the following items are present in the figure legend, table legend, main text, or Methods section.

n/a	Confirmed	
☐	☒	The exact sample size (*n*) for each experimental group/condition, given as a discrete number and unit of measurement
☐	☒	A statement on whether measurements were taken from distinct samples or whether the same sample was measured repeatedly
☐	☒	The statistical test(s) used AND whether they are one- or two-sided<br>*Only common tests should be described solely by name; describe more complex techniques in the Methods section.*
☐	☒	A description of all covariates tested
☐	☒	A description of any assumptions or corrections, such as tests of normality and adjustment for multiple comparisons
☐	☒	A full description of the statistical parameters including central tendency (e.g. means) or other basic estimates (e.g. regression coefficient) AND variation (e.g. standard deviation) or associated estimates of uncertainty (e.g. confidence intervals)
☐	☒	For null hypothesis testing, the test statistic (e.g. *F*, *t*, *r*) with confidence intervals, effect sizes, degrees of freedom and *P* value noted<br>*Give P values as exact values whenever suitable.*
☒	☐	For Bayesian analysis, information on the choice of priors and Markov chain Monte Carlo settings
☒	☐	For hierarchical and complex designs, identification of the appropriate level for tests and full reporting of outcomes
☒	☐	Estimates of effect sizes (e.g. Cohen's *d*, Pearson's *r*), indicating how they were calculated

Our web collection on statistics for biologists contains articles on many of the points above.

Software and code

Policy information about availability of computer code

Data collection	NanoScope Version 9.1, LAS X Version 3.5, QuantStudio Design & Analysis Desktop Software were used to acquire data.
Data analysis	GraphPad Prism Version 8.2 were used to realize data visualization.<br>Micro-CT images were analysed with 3D post-processing workstation Inveon Research Workplace V 2.2.0.<br>The KPFM results were analyzed with NanoScope Analysis 1.5.<br>The XRD results were analyzed with MDI Jade Version 6.5.<br>Weatern blot result were analyzed by using an Image Lab Version 6.0 analysis software.<br>Statistical analysis was performed using SPSS-21.0 (International Business Machines Corporation (IBM), USA) software.<br>Part of the schematic was produced in the website BioRender.com.

For manuscripts utilizing custom algorithms or software that are central to the research but not yet described in published literature, software must be made available to editors and reviewers. We strongly encourage code deposition in a community repository (e.g. GitHub). See the Nature Portfolio guidelines for submitting code & software for further information.

Data

Policy information about availability of data

All manuscripts must include a data availability statement. This statement should provide the following information, where applicable:

- Accession codes, unique identifiers, or web links for publicly available datasets
- A description of any restrictions on data availability
- For clinical datasets or third party data, please ensure that the statement adheres to our policy

All relevant data supporting the key findings of this study are available within the article and the Supplementary Information file. Source data are provided with this paper.

Research involving human participants, their data, or biological material

Policy information about studies with human participants or human data. See also policy information about sex, gender (identity/presentation), and sexual orientation and race, ethnicity and racism.

Reporting on sex and gender	Not applicable.
Reporting on race, ethnicity, or other socially relevant groupings	Not applicable.
Population characteristics	Not applicable.
Recruitment	Not applicable.
Ethics oversight	Not applicable.

Note that full information on the approval of the study protocol must also be provided in the manuscript.

Field-specific reporting

Please select the one below that is the best fit for your research. If you are not sure, read the appropriate sections before making your selection.

☒ Life sciences ☐ Behavioural & social sciences ☐ Ecological, evolutionary & environmental sciences

For a reference copy of the document with all sections, see nature.com/documents/nr-reporting-summary-flat.pdf

Life sciences study design

All studies must disclose on these points even when the disclosure is negative.

Sample size	Because animal experiments used homologous control, we used the minimum number of animals (n≥3) necessary to achieve statistical significance. In cell experiments, 3 biological independent samples in each group were used for detection in different experiments to meet the statistical requirements. The detailed sample size for each experiment is shown in all the figure legends.
Data exclusions	We exclude animals that unexpectedly died during the course of the experiment.
Replication	Once instruments and protocols were established, all attempts at replication were successfully reproduced. All data were replicated at least 3 times.
Randomization	Animals were allocated randomly into the different experimental group. In cell experiments, before being inoculated to the surface of materials in different groups, cells need to be digested from culture bottles to prepare cell suspensions uniformly. The process of adding cells into different groups is equal and random.
Blinding	Authors were not blinded because different materials were grouped prior to experiments and analysis. The objective was to compare the effects of different groups in parallel. Stated in the methods section.

Reporting for specific materials, systems and methods

We require information from authors about some types of materials, experimental systems and methods used in many studies. Here, indicate whether each material, system or method listed is relevant to your study. If you are not sure if a list item applies to your research, read the appropriate section before selecting a response.

Materials & experimental systems

n/a	Involved in the study
☐	☒ Antibodies
☒	☐ Eukaryotic cell lines
☒	☐ Palaeontology and archaeology
☐	☒ Animals and other organisms
☒	☐ Clinical data
☒	☐ Dual use research of concern
☒	☐ Plants

Methods

n/a	Involved in the study
☒	☐ ChIP-seq
☒	☐ Flow cytometry
☒	☐ MRI-based neuroimaging

Antibodies

Antibodies used

Antibodies used in Western Blot: anti-RUNX2 (ab76956, 1:1000, diluted with 5% w/v skimmed milk), anti-BMP2 (ab214821, 1:1000, diluted with 5% w/v skimmed milk) and anti-Osteopontin (ab63856, 1:1000, diluted with 5% w/v skimmed milk) were purchased from Abcam. The primary antibody anti-β-actin (AF0003, 1:1000, diluted with 5% w/v skimmed milk) and secondary antibody HRP-labeled IgG (A0208, A0216, 1:1000, diluted with TBST) were purchased from Beyotime, China.
Antibodies used in immunofluorescence: polyclonal rabbit anti-RUNX2 (1:100, diluted with 3% (w/v) BSA solution; Abcam, ab114133), goat anti-rabbit IgG H&L Alexa Fluor488 (1:200, diluted with PBS, Abcam, ab150081).

Validation

Anti-RUNX2, ab76956, key features and details: Mouse monoclonal [2B9] to RUNX2; Suitable for: ICC/IF, Flow Cyt, WB; Reacts with: Rat, Human, Recombinant fragment; Isotype: IgG2a. https://www.abcam.cn/products/primary-antibodies/runx2-antibody-2b9-ab76956.html

Anti-BMP2, ab214821, Key features and details: Produced recombinantly (animal-free) for high batch-to-batch consistency and long term security of supply; Rabbit monoclonal [EPR20807] to BMP2; Suitable for: Flow Cyt (Intra), WB, ICC/IF; Reacts with: Mouse, Rat, Human. https://www.abcam.cn/products/primary-antibodies/bmp2-antibody-epr20807-ab214821.html

Anti-Osteopontin, ab63856, Key features and details: Rabbit polyclonal to Osteopontin; Suitable for: ICC/IF, IHC-P, WB; Reacts with: Mouse, Rat, Human; Isotype: IgG. https://www.abcam.cn/products/primary-antibodies/osteopontin-antibody-ab63856.html

Anti-β-actin, AF0003, Beyotime: https://www.beyotime.com/product/AF0003.htm

HRP-labeled IgG, A0208, A0216: https://www.beyotime.com/product/A0208.htm; https://www.beyotime.com/product/A0216.htm

Polyclonal rabbit anti-RUNX2, Key features and details: Rabbit polyclonal to RUNX2; Suitable for: ICC/IF, WB; Reacts with: Human, Mouse, Rat, Rabbit; Isotype: IgG. https://www.abcam.cn/products/primary-antibodies/runx2-antibody-ab114133.html

Goat anti-rabbit IgG H&L Alexa Fluor488, ab150081, Key features and details: Goat Anti-Rabbit IgG H&L (Alexa Fluor® 488) preadsorbed; Conjugation: Alexa Fluor® 488. Ex: 495nm, Em: 519nm; Host species: Goat; Isotype: IgG; Suitable for: IHC-Fr, ICC/IF, Flow Cyt, IHC-P, ELISA. https://www.abcam.cn/products/secondary-antibodies/goat-rabbit-igg-hl-alexa-fluor-488-preadsorbed-ab150081.html

Animals and other research organisms

Policy information about studies involving animals; ARRIVE guidelines recommended for reporting animal research, and Sex and Gender in Research

Laboratory animals	7-week-old, 200-220 g, male Sprague-Dawley (SD) rats were used in this study.
Wild animals	The study did not involve wild animals.
Reporting on sex	In order to exclude gender variable factors in animal testing, single-sex experimental animals were used in this study.
Field-collected samples	The study did not involve samples collected from the field.
Ethics oversight	Animal licenses were approved by the Animal Care and Use Committee of Peking University (IACUC number: LA2021230).

Note that full information on the approval of the study protocol must also be provided in the manuscript.
